# The mediating role of psychological resilience and loneliness between physical activity and mobile phone addiction among adolescents

**DOI:** 10.3389/fnut.2026.1761917

**Published:** 2026-03-19

**Authors:** Xiaofen Ding, Wanlin Liu

**Affiliations:** 1 Hunan First Normal University, Hunan, China; 2 College of Art and Education, Hunan Foreign Language Vocational College, Hunan, China

**Keywords:** chain mediation, loneliness, mobile phone addiction, physical activity, psychological resilience

## Abstract

**Objective:**

To explore the relationship between physical activity and mobile phone addiction in adolescents and further explore the mediating chain effect of psychological resilience and loneliness.

**Methods:**

1,226 adolescents were investigated by an International physical activity questionnaire, mobile phone addiction scale, Psychological resilience scale, and Loneliness scale.

**Results:**

There were significant gender differences in adolescents psychological resilience and mobile phone addiction (*p* < 0.01). Physical activity was negatively correlated with mobile phone addiction (*r* = −0.21, *p* < 0.01) and loneliness (*r* = −0.22, *p* < 0.01), physical activity was positively correlated with psychological resilience (*r* = 0.42, *p* < 0.01), psychological resilience was negatively correlated with loneliness (*r* = −0.26, *p* < 0.01), respectively was negatively correlated with mobile phone addiction (*r* = −0.23, *p* < 0.01), and loneliness was positively correlated with mobile phone addiction (*r* = 0.32, *p* < 0.01). Psychological resilience had a significant mediating effect between physical activity and mobile phone addiction, loneliness had a significant mediating effect between physical activity and mobile phone addiction, and psychological resilience and loneliness had a significant chain mediating effect between moderate and high physical activity and mobile phone addiction.

**Conclusion:**

(1) Physical activity positively predicts phone addiction; (2) Physical activity can not only directly affect mobile phone addiction but also affect mobile phone addiction through the mediating effects of psychological resilience and loneliness respectively; (3) Physical activity can also influence mobile phone addiction through the chain mediating effect of psychological resilience and loneliness. These findings can be directly applied by integrating structured physical activity programs into school schedules to enhance adolescents’ psychological resilience against addictive behaviors.

## 1 Introduction

Mobile Phone Addiction (MPA), also known as mobile phone dependence, problematic mobile phone use, or nomophobia, refers to a maladaptive psychological or behavioral state of addiction in individuals regarding mobile phone use (1). Mobile phone addiction not only leads to environmentally inappropriate phone use (e.g., using a phone while driving), fear of losing one’s phone, and disruptions to daily life (2), but it also often negatively impacts individuals’ physical and mental health, such as reducing sleep quality (2) and impairing cognitive function (3). The Self-Determination Theory suggests that relationships, autonomy, and competence are the three fundamental aspects of individuals’ psychological needs. When these basic psychological needs are obstructed, compensatory motivations may develop (4). Adolescents, as a primary demographic of mobile phone users, are more prone to mobile phone addiction compared to other adult groups due to their weaker self-control and regulation abilities. When facing psychological issues, they are more likely to escape reality through mobile phone use, leading to mobile phone addiction (5). Currently, mobile phone addiction among addiction has become a focus of societal attention, and exploring its influencing factors has become an important research topic for scholars both domestically and internationally. However, most existing studies focus on the impact of single factors on mobile phone addiction, leaving room for deeper exploration.

Physical activity is defined as “any bodily movement produced by skeletal muscles that results in energy expenditure” (6). Appropriate physical activity provides numerous health benefits, including controlling obesity, preventing cardiovascular diseases, and alleviating symptoms of negative emotions. The World Health Organization (WHO) recommends that adults engage in at least 150–300 min of moderate-intensity physical activity or at least 75–150 min of vigorous-intensity physical activity per week (7). In addition to being influenced by individual characteristics [e.g., emotions (5), cognition (6)] and environmental factors [e.g., social support (8), physical exercise atmosphere (9)], mobile phone addiction has recently garnered attention for its relationship with physical activity. A growing body of research is dedicated to exploring this relationship. Physical activity is also a significant factor influencing mobile phone addiction among young people (10). Relevant studies show that adolescents’ physical activity levels significantly and negatively predict mobile phone addiction (11). Other research has found a significant negative correlation between adolescents’ physical activity and mobile phone addiction, with mobile phone addiction scores decreasing as physical activity levels increase (12). This study also found that the impact of physical activity on mobile phone addiction can be moderated by exercise type. A review suggests that low levels of physical activity may contribute to mobile phone addiction. In summary, while valuable findings have been made regarding the relationship between physical activity and mobile phone addiction, they remain insufficient. The potential influencing factors between adolescents’ physical activity and mobile phone addiction still need exploration, and the mediating effects between the two require further investigation.

Recent research on mobile phone addiction has begun to focus on the protective role of psychological resilience. The concept of “resilience” was first applied in physical science, referring to the ability of a physical system to return to its original state after external disturbances (12). Later, the American Psychological Association (APA) defined psychological resilience as the ability to recover and adapt to adversity, trauma, threats, or sources of stress (13). Previous studies have demonstrated that physical activity of different intensities and durations is positively correlated with psychological resilience (14). Research confirms that adolescents who meet the WHO’s recommended physical activity levels exhibit better psychological resilience than those who do no (15). Scholars have proposed that physical activity is an important variable influencing adolescents’ psychological resilience levels, positively affecting resilience. As physical activity intensity increases, psychological resilience significantly improves (15). Physical activity is an effective way to cultivate psychological resilience in adolescents and can enhance their problem-solving abilities, interpersonal skills, self-confidence, emotional regulation, and cognitive abilities (14). The self-regulatory failure model suggests that individuals with psychosocial problems may struggle to control their mobile phone use due to insufficient self-control and regulation abilities (16). High levels of psychological resilience promote positive psychological and behavioral development in adolescents, such as stronger self-control and restraint, enabling them to regulate their mobile phone use (17). In contrast, adolescents with low psychological resilience may resort to escapism and excessive internet use when facing negative life events, leading to mobile phone addiction (18). In summary, physical activity may increase adolescents’ psychological resilience levels, thereby regulating their inappropriate mobile phone use and mitigating mobile phone addiction. Thus, psychological resilience may serve as a crucial bridge between physical activity and mobile phone addiction. Therefore, we propose: H1: Psychological resilience mediates the relationship between physical activity and mobile phone addiction among adolescents.

Loneliness, also referred to as isolation, separation, or a lack of belonging, is a negative emotion arising from the discrepancy between an individual’s desire for social interaction and their actual level of social engagement (19). It reflects the difference between one’s actual and desired social relationships. Loneliness has become a significant risk factor affecting adolescents’ daily lives. Adolescents with high levels of loneliness tend to excessively immerse themselves in their own world, while physical activity provides an alternative environment. A longitudinal study found that increased physical activity reduces loneliness levels, highlighting the positive role of physical activity in alleviating loneliness (20). Additionally, individuals with high physical activity levels exhibit lower loneliness and higher wellbeing compared to those with low physical activity levels (21). Physical activities, such as walking, running, participating in sports clubs, and attending physical education classes, can connect adolescents’ lives, making their interactions more active and close, thereby reducing loneliness (22). On the other hand, scholars believe that loneliness and mobile phone addiction are closely linked. Individuals with high loneliness may overuse smartphones, leading to mobile phone addiction (23). Loneliness, as a negative emotion, positively predicts mobile phone addiction. The compensatory internet use theory suggests that when individuals face psychological problems in the real world, they may turn to powerful smartphones to escape pain, resulting in mobile phone addiction (24). From the above, it is evident that physical activity can influence loneliness, while loneliness can also predict the development of mobile phone addiction. Therefore, Adolescents’ physical activity may indirectly affect mobile phone addiction through loneliness. Thus, we Propose: H2: Loneliness mediates the relationship between physical activity and mobile phone addiction among adolescents (25).

**Figure 1:**
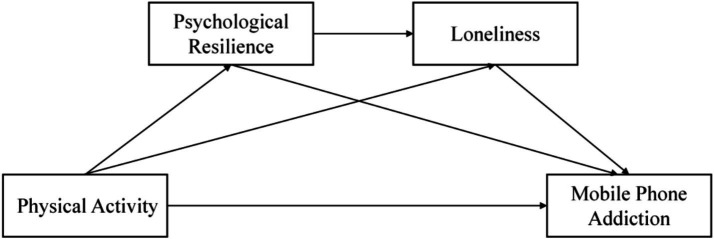
Research hypothesis model.

Current lifestyles have reduced face-to-face interactions among adolescents, potentially exacerbating perceived loneliness (26). Psychological resilience, as an important psychological resource, helps adolescents mitigate the impact of negative emotions and adapt to adversity when facing emotional challenges (26). According to the compensatory model of psychological resilience, resilience has positive functions, alleviating loneliness caused by social anxiety and helping adolescents make positive subjective judgments about their life conditions (27). Individuals with high psychological resilience can better regulate themselves in complex interpersonal situations, experiencing lower levels of loneliness (28). Research also finds that psychological resilience negatively predicts individuals’ loneliness levels (29). In summary, while physical activity provides a new perspective for studying the formation and development of mobile phone addiction, beyond the individual mediating roles of psychological resilience and loneliness in the relationship between adolescents’ physical activity and mobile phone addiction, there may also be a serial mediation effect between the two. Therefore, we propose: H3: Psychological resilience and loneliness serially mediate the relationship between adolescents’ physical activity and mobile phone addiction. The hypothesized path is shown in Figure 1.

## 2 Methods

We followed the principle of convenience sampling and conducted a cross-sectional survey of adolescents from two high school in Hunan Province between March 28, 2025, and May 10, 2025. This research has been approved by the Ethics Committee of Hunan First Normal University. To ensure sufficient statistical power, the sample size for this study was determined using G*Power software (version 3.0.3). The analysis was performed by selecting “*F* tests—Linear multiple regression: Fixed model. With an effect size *f* of 0.25, a significance level *α* of 0.05, and a statistical power (1–*β*) of 0.95, the minimum required sample size was calculated to be 210 participants. We collected the questionnaires through an online voluntary registration process, and the participants were under 18 years old, and we provided written informed consent to their parents (or legal guardians) before taking part in the study. A total of 1,325 questionnaires were distributed, and 1,325 were returned. After excluding some invalid questionnaires, we obtained 1,226 valid questionnaires from adolescents, resulting in an effective response rate of 92.53%. Among the surveyed students, 567 were male, and 659 were female; The average age was (17.21 ± 1.85) years.

### 2.1 Research tools

1) Psychological resilience

The Connor–Davidson Resilience Scale (CD-RISC), developed by Connor and Davidson (30) and revised by Xin and Zhang (31), was used. This scale consists of 25 items divided into three dimensions, such as “I can see the humorous side of things” (optimism), “I can handle whatever comes my way” (tenacity), and “I feel in control of my life” (strength). A 5-point Likert scale was used (1 = never, 5 = always), and the scores of all items were summed to obtain the total psychological resilience score. Higher scores indicate stronger psychological resilience. The internal consistency (Cronbach’s *α*) of the scale was 0.84.

2) Loneliness

The UCLA Loneliness Scale (UCLA), developed by Russell, was used to assess individuals’ loneliness resulting from the discrepancy between desired and actual social interactions (32). This scale consists of 20 items, scored on a 4-point scale (1 = never, 4 = often). Items 1, 5, 6, 9, 10, 15, 16, 19, and 20 were reverse-scored. The scores of all items were summed to obtain the total loneliness score, with higher scores indicating greater loneliness. The internal consistency (Cronbach’s *α*) of the scale was 0.83.

3) Mobile phone addiction

The Mobile Phone Addiction Index (MPAI), developed by Leung (33), was used. This scale consists of 17 items, scored on a 5-point Likert scale (1 = not at all, 5 = very often). The scale includes four dimensions, such as “You always feel that the time spent using your mobile phone is insufficient” (loss of control), “You feel anxious about missing calls when you do not check your phone for a certain period” (withdrawal), “You use your mobile phone to communicate with others when you feel lonely” (escape), and “Sometimes you prefer using your mobile phone over handling other more urgent matters” (inefficiency). Higher scores indicate a higher degree of mobile phone addiction. The internal consistency (Cronbach’s *α*) of the scale was 0.91.

4) Physical activity

The International Physical Activity Questionnaire-Short Form (IPAQ-SF), developed by Meeus et al. (34), was used. This questionnaire consists of 7 items, with 6 items assessing physical activity and 1 item assessing sedentary time. Participants were asked to report activities lasting at least 10 min over the past 7 days. Physical activity over the past 7 days was measured by the time spent in three activity intensities (light, moderate, and vigorous), with metabolic equivalent (MET) values assigned to each intensity: 3.3 for light activities (e.g., walking), 4.0 for moderate activities, and 8.0 for vigorous activities. The total energy expenditure was calculated by multiplying the time spent in each activity by its corresponding MET value.

### 2.2 Data processing and analysis

The collected data were processed using Excel software for value assignment and reverse scoring. Invalid questionnaires, such as those with uniform responses, patterned responses, or completion times below 150 s or above 1,800 s, were excluded. The data were then imported into SPSS 24.0 for statistical analysis. Descriptive statistics were performed using SPSS. Reliability analysis was conducted using the Alpha model. Common method bias was tested using Harman’s single-factor test. Independent sample *t*-tests were used to examine variable differences between genders. Pearson correlation analysis was used to calculate correlations between variables. The SPSS PROCESS macro was used to test the serial mediation effects of psychological resilience and loneliness between physical activity and mobile phone addiction. A significance level of *p* < 0.05 was set.

## 3 Results

### 3.1 Common method bias test

To control for common method bias, Harman’s single-factor test was conducted. Exploratory factor analysis (EFA) was performed on all scale items (excluding demographic variables). A total of 15 factors with eigenvalues greater than 1 were extracted, and the first factor explained 20.99% of the variance, which is below the critical threshold of 40%. This indicates that there was no significant common method bias in this study (35).

### 3.2 Descriptive statistics of survey variables

Table 1 presents the means and standard deviations of age, physical activity, psychological resilience, loneliness, and mobile phone addiction. To examine whether there were differences in the study variables between genders, independent samples *t*-tests were conducted with gender as the grouping variable and age, physical activity, psychological resilience, loneliness, and mobile phone addiction as the test variables. The results revealed significant differences in age, psychological resilience, and mobile phone addiction between genders, while no significant differences were found in physical activity and loneliness between genders.

**Table 1:** Gender differences in study variables.

Variable	Total (*n* = 1,226)	Male (*n* = 567)	Female (*n* = 659)	*T*	*P*
	*M* ± SD	*M* ± SD	*M* ± SD		
Age	17.21 ± 1.85	17.36 ± 1.77	17.09 ± 1.90	2.53^*^	0.01
Physical activity	3,686.46 ± 2,884.58	3,837.37 ± 3,000.32	3,556.62 ± 2,776.88	1.70	0.09
Psychological resilience	75.14 ± 18.48	77.83 ± 18.92	72.81 ± 17.76	4.79^**^	0.00
Loneliness	47.88 ± 8.09	47.76 ± 8.06	47.89 ± 8.12	−0.49	0.63
Mobile phone addiction	41.64 ± 12.62	40.68 ± 12.36	42.45 ± 12.80	−2.46^**^	0.01

^*^P < 0.05, ^**^P < 0.01, *M*, Mean; SD, Standard Deviation.

### 3.3 Correlation analysis results of physical activity, psychological resilience, loneliness, and mobile phone addiction

Partial correlation analysis, controlling for gender and age, revealed the following results (Table 2): Physical activity was significantly positively correlated with psychological resilience (*r* = 0.42, *p* < 0.001), while it was significantly negatively correlated with loneliness (*r* = −0.22, *p* < 0.001) and mobile phone addiction (*r* = −0.21, *p* < 0.001). Psychological resilience was significantly negatively correlated with loneliness (*r* = −0.26, *p* < 0.001) and mobile phone addiction (*r* = −0.23, *p* < 0.001). Loneliness was significantly positively correlated with mobile phone addiction (*r* = 0.32, *p* < 0.001).

**Table 2:** Correlation analysis of study variables.

Variable	Physical activity	Physical activity	Physical activity	Physical activity
Physical activity	–			
Psychological resilience	0.42^**^	–		
Loneliness	−0.22^**^	−0.26^**^	–	
Mobile phone addiction	−0.21^**^	−0.23^**^	0.32^**^	–

^*^P < 0.05, ^**^P < 0.01.

### 3.4 Mediation effects of psychological resilience and loneliness on the relationship between physical activity and mobile phone addiction

In the mediation analysis model of this study, physical activity was the independent variable, mobile phone addiction was the dependent variable, and gender and age were control variables. The mediating roles of psychological resilience and loneliness were examined separately. The SPSS PROCESS macro developed by Hayes (55) was used, and Model 6 was selected. According to the regression analysis results in (Table 3): Physical activity significantly and positively predicted psychological resilience (*β* = 0.40, *p* < 0.001), while it significantly and negatively predicted loneliness (*β* = −0.14, *p* < 0.001) and mobile phone addiction (*β* = −0.10, *p* < 0.001). Psychological resilience significantly and negatively predicted loneliness (*β* = −0.20, *p* < 0.001) and mobile phone addiction (*β* = −0.12, *p* < 0.001). Loneliness significantly and positively predicted mobile phone addiction (*β* = 0.27, *p* < 0.001).

**Table 3:** Regression analysis results of the study variables.

Regression equation			Overall fit indices		Regression coefficient significance	
Outcome variable	Predictor variable	*R*	*R* ^2^	*F*	*β*	*t*
Psychological resilience	Gender	0.43	0.18	93.22^**^	−0.12	−4.55^**^
	Age				−0.03	−1.14
	Physical activity				0.40	15.86^**^
Loneliness	Gender	0.29	0.08	27.78^**^	−0.02	−0.65
	Age				0.04	1.29
	Physical activity				−0.14	−4.56^**^
	Psychological resilience				−0.20	−6.68^**^
Mobile phone addiction	Gender	0.37	0.14	40.20^**^	0.04	1.52
	Age				−0.06	−2.46^*^
	Physical activity				−0.10	−3.34^**^
	Psychological resilience				−0.12	−3.88^**^
	Loneliness				0.27	9.79^**^

^*^P < 0.05, ^**^P < 0.01.

**Figure 2:**
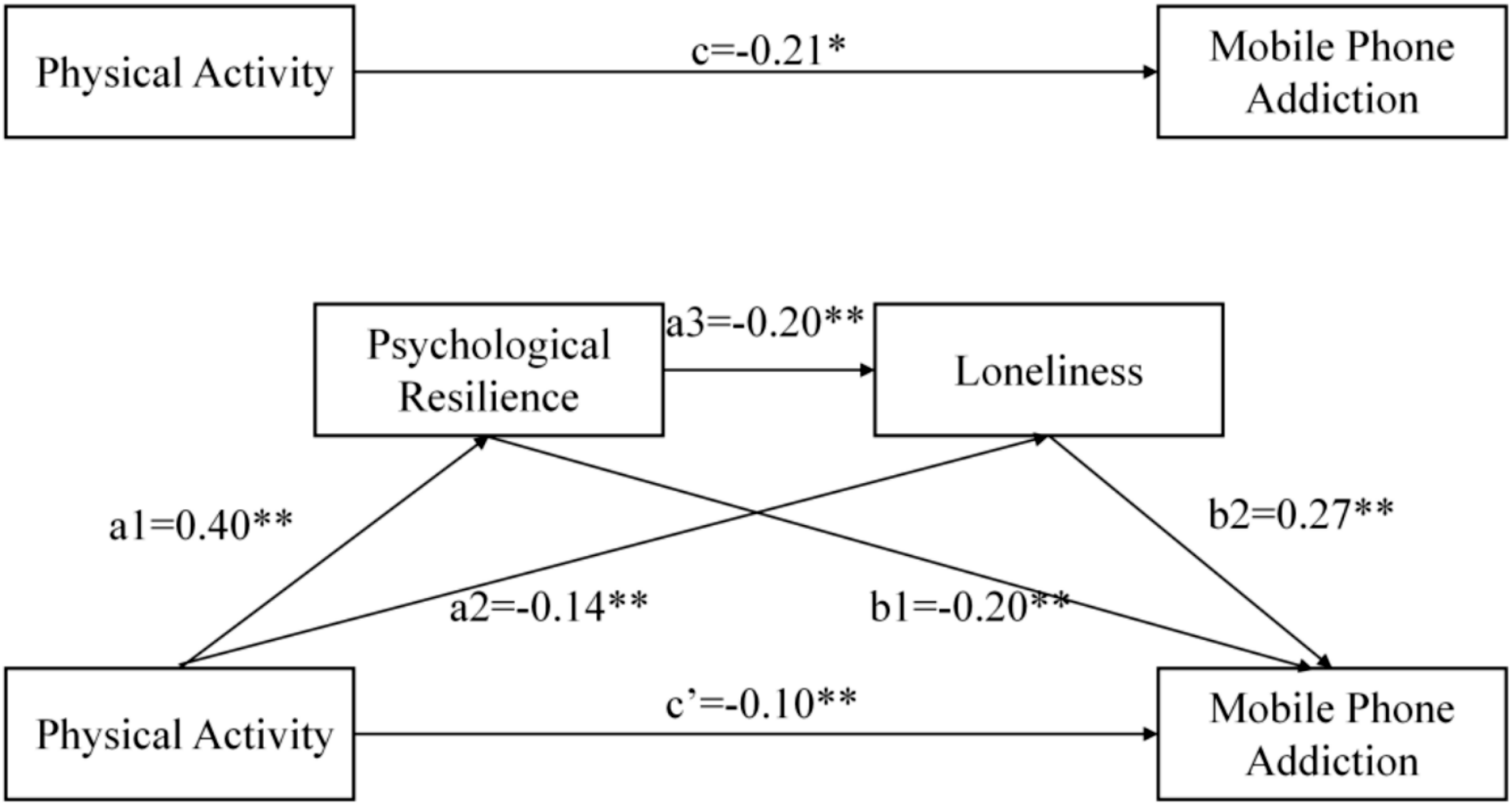
Chain mediation model.

The bias-corrected nonparametric percentile Bootstrap method was used, with 5,000 repeated samples and a default confidence interval of 95%. The results in (Table 4) showed: The mediating effect of physical activity on mobile phone addiction through psychological resilience was (*β* = −0.05, 95% CI: [−0.0747, −0.0215]). The interval did not include “0,” indicating a significant mediating effect, accounting for 23.1% of the total effect. The mediating effect of physical activity on mobile phone addiction through loneliness was (*β* = −0.04, 95% CI: [−0.0574, −0.0182]). The interval did not include “0,” indicating a significant mediating effect, accounting for 18.1% of the total effect. The chain mediating effect of physical activity on mobile phone addiction through psychological resilience and loneliness was (*β* = −0.02, 95% CI: [−0.0325, −0.0139]). The interval did not include “0,” indicating a significant mediating effect, accounting for 10.9% of the total effect. The total mediating effect of physical activity on mobile phone addiction was (*β* = −0.11, 95% CI: [−0.1386, −0.0760]). The interval did not include “0,” indicating a significant total mediating effect, accounting for 52.4% of the total effect. The direct effect of physical activity on mobile phone addiction was (*β* = −0.10, 95% CI: [−0.0007, −0.0002]). The interval did not include “0,” indicating a significant direct effect, accounting for 47.6% of the total effect. The total effect of physical activity on mobile phone addiction was (*β* = −0.21, 95% CI: [−0.0011, −0.0007]). The interval did not include “0,” indicating a significant total effect. The total mediation effect accounted for 52.4% of the total effect, indicating that the majority of physical activity’s influence on mobile phone addiction operates through psychological and social–emotional mechanisms rather than direct effects. Based on these findings, a chain mediation model was constructed to visually illustrate the intrinsic relationships among the variables, as shown in Figure 2.

**Table 4:** Mediating effect test results.

Effect path	*β*	BootSE	95%CI	Relative proportion of intermediaries
Physical activity ⇒ Psychological resilience ⇒ Mobile phone addiction	−0.05	0.0136	[−0.0747, −0.0215]	23.8%
Physical activity ⇒ Loneliness ⇒ Mobile phone addiction	−0.04	0.0100	[−0.0574, −0.0182]	19.0%
Physical activity ⇒ Psychological resilience ⇒ Loneliness ⇒ Mobile phone addiction	−0.02	0.0047	[−0.0325, −0.0139]	9.5%
Total mediation effect	−0.11	0.0160	[−0.1386, −0.0760]	52.4%
Direct effect	−0.10	0.0001	[−0.0007, −40.0002]	47.6%
Total effect	−0.21	0.0001	[−0.0011, −0.0007]	100%

## 4 Discussion

This study, using a sample of 1,226 *adolescents, re-examined* the relationship between physical activity and mobile phone addiction, further validating the chain mediation effects of psychological resilience and loneliness. It provides new evidence for the intrinsic connection between physical activity and mobile phone addiction, clarifying the relationship between the two. The mediation effects were achieved through three pathways: (1) the independent mediating role of psychological resilience; (2) the independent mediating role of loneliness; and (3) the chain mediating role of psychological resilience and loneliness. Hypotheses H1, H2, and H3 were all confirmed.

Independent samples *t*-tests revealed significant gender differences in psychological resilience and mobile phone addiction among adolescents. Compared to female students, male students exhibited higher levels of psychological resilience and lower levels of mobile phone addiction (36). When facing stressful or challenging situations, male students tend to actively confront problems and adopt a rational approach, while female students are more likely to respond emotionally. Societal expectations often portray males as stronger, more restrained, and composed, whereas females are more readily understood and forgiven for emotional expression. As a result, females are more inclined to seek external support when encountering difficulties, while males tend to face challenges independently, which helps enhance their psychological resilience. Previous research has identified gender as a significant demographic indicator of mobile phone addiction among adolescents, with female students exhibiting more severe addiction. When experiencing high levels of negative emotions, males demonstrate stronger adaptability and are more likely to overcome challenges independently, rarely relying on mobile phones to vent or express their feelings (37). In contrast, females are more accustomed to expressing and venting emotions, often using mobile phones for social interaction or entertainment to alleviate negative emotions, which increases their dependency on mobile phones (38, 39). Additionally, females tend to have stronger emotional engagement when using mobile phones, and the various applications on mobile devices serve as tools for emotional expression, making them more prone to dependency than males.

The Chinese digital environment may further interact with these cultural and gendered expectations. Popular platforms such as WeChat and Douyin/TikTok are deeply embedded in adolescents’ daily lives, providing not only entertainment but also key channels for social interaction, self-presentation, and emotional expression. Female adolescents may rely more on these platforms to maintain peer networks, share daily experiences, and seek emotional support, thereby integrating mobile phone use into their core social and emotional routines (40). In the context of a collectivist culture, where belonging and connectedness are highly valued, this reliance can intensify the subjective importance of staying constantly connected and responsive, which may increase vulnerability to mobile phone addiction. In contrast, male adolescents may use these platforms more instrumentally (e.g., obtaining information, coordinating activities) or for achievement-oriented entertainment (e.g., competitive games), which may involve high usage but does not necessarily translate into the same level of emotional dependency (41). Taken together, these culturally situated patterns of socialization, educational pressure, and technology use provide a more nuanced explanation of why male students in this Chinese sample showed higher psychological resilience and lower mobile phone addiction than female students, and suggest that observed gender differences should be interpreted as products of sociocultural processes rather than fixed, biological differences.

This study found that physical activity negatively predicts mobile phone addiction among adolescents, consistent with previous research indicating that higher levels of physical activity are associated with lower levels of mobile phone addiction (14, 42–45). According to the health behavior theory in exercise psychology, individual behavior is influenced not only by psychological factors but also by external environmental factors, with the latter playing a more decisive role (38). Bandura’s triadic reciprocal determinism suggests that environment, individual, and behavior interact with each other (46). As an important external environmental stimulus, physical activity not only improves physical health but also significantly impacts mental health and social adaptation. Neurophysiological mechanisms can also explain the effect of physical activity on mobile phone addiction. Inhibitory control is closely related to addictive behaviors, and deficits in inhibitory control are a key factor in mobile phone addiction (47, 48). Individuals with higher levels of mobile phone addiction often exhibit poorer inhibitory control. Physical activity is an effective intervention for improving inhibitory control. Studies have shown that 30 min of acute physical activity significantly enhances performance in Go/no-go tasks, which require inhibitory control (49). Regular long-term physical activity interventions also improve task performance requiring high levels of inhibitory control. Furthermore, physical activity reduces screen time and sedentary behavior among adolescents, decreasing their dependency on mobile phones. It also alleviates withdrawal symptoms, loss of control, and negative emotions such as anxiety, depression, and loneliness in individuals with mobile phone addiction (9). In summary, physical activity brings positive benefits in reducing mobile phone addiction among adolescents, likely through its favorable psychological effects. Therefore, promoting physical activity is a feasible approach to mitigating mobile phone addiction.

The mediation analysis revealed that psychological resilience mediates the relationship between physical activity and mobile phone addiction, confirming Hypothesis H1. First, physical activity positively influences psychological resilience (50). It enhances emotional regulation by strengthening specific brain regions and neural circuits, thereby improving psychological resilience. It also fosters optimism and social support, further boosting psychological resilience (51). Second, according to the dynamic model of resilience, psychological resilience is an innate ability that enables individuals to regulate negative emotions and avoid setbacks caused by real-life challenges (51). High school life, as a transitional period to adulthood, involves significant stress and challenges in daily life, interpersonal relationships, and academics. Students often turn to mobile phones to alleviate negative emotions, leading to dependency and addiction. Specifically, students with high psychological resilience adopt proactive coping strategies, overcoming difficulties and reducing reliance on mobile phones. Thus, physical activity can reduce mobile phone addiction by enhancing psychological resilience.

Loneliness also plays a significant mediating role in the relationship between physical activity and mobile phone addiction, confirming Hypothesis H2. The distraction hypothesis posits that physical activity diverts attention from negative emotions, allowing individuals to regulate their feelings, emotions, and behaviors effectively. Additionally, physical activity stimulates endorphin secretion, promoting feelings of happiness and wellbeing, thereby reducing loneliness (52). Furthermore, students experiencing loneliness often lack social interactions and become dependent on mobile phones due to the engaging and entertaining nature of mobile applications (53). Physical activity facilitates social interaction, reducing loneliness and dependency on mobile phones. In summary, physical activity can lower loneliness levels by enhancing social support, diverting negative emotions, or promoting endorphin release, thereby mitigating mobile phone addiction.

Physical activity among adolescents also influences mobile phone addiction through the chain mediation of psychological resilience and loneliness, representing an important pathway. This finding aligns with previous research indicating that psychological resilience significantly affects loneliness (54). Students with high psychological resilience not only adopt strategies such as “changing perspectives” to alleviate loneliness but also effectively manage negative environmental influences (38). For example, they reduce social avoidance and distress in social settings, fostering positive interpersonal relationships and lowering loneliness. Additionally, students with lower loneliness levels are better at controlling emotions and reducing negative emotional experiences (38). Consequently, they are less likely to rely on mobile phones to cope with negative emotions, reducing the risk of developing mobile phone addiction (54).

### 4.1 Limitations

This study provides an in-depth exploration of the impact of physical activity on mobile phone addiction among adolescents. The results of correlation and mediation analyses support the hypotheses, offering empirical evidence for the mechanisms underlying the relationship between physical activity and mobile phone addiction. The findings have practical implications for the prevention and control of mobile phone addiction among adolescents. Future interventions should consider both psychological factors and physical activity levels to achieve optimal outcomes. However, this study has some limitations. First, as a cross-sectional study, it cannot establish causal relationships between physical activity and mobile phone addiction. Longitudinal studies are needed to validate these causal relationships. Second, variations in participant characteristics and measurement tools may introduce errors, warranting further investigation into the relationship between physical activity and mobile phone addiction.

## 5 Conclusion

Increasing the level of physical activity in adolescents may help prevent and reduce mobile phone addiction behavior, and this effect may be partly due to physical activity improving individuals’ levels of psychological resilience and loneliness. These findings have potential applications for the development of targeted interventions. Further research should focus on designing tailored intervention protocols that combine physical exercise with psychological support to maximize protective effects across diverse adolescent populations.

## Data Availability

The original contributions presented in the study are included in the article/supplementary material, further inquiries can be directed to the corresponding author.
